# Emergency packets handing in queue aware congestion avoidance schemes in IoHT

**DOI:** 10.1371/journal.pone.0352502

**Published:** 2026-07-14

**Authors:** Muhammad Zafarullah, Ata Ullah, Sajjad Ahmed Ghauri, Ghalib Muhammad Waqas Janjua

**Affiliations:** 1 Department of Computer Science, National University of Modern Languages, Islamabad, Pakistan; 2 School of Engineering & Applied Sciences, Isra University, Islamabad, Pakistan; 3 Higher Colleges of Technology, Abu Dhabi; Vignans Institute of Information Technology, INDIA

## Abstract

The Internet of Healthcare Things (IoHT) emerged as an enabling technology that is strongly required for real-time patient monitoring and emergency healthcare services. However, the massive number of messages sent from heterogeneous sensors during the emergency situations generally leads to congestion in the network, causing it to overflow the buffer and lose some vital packets. Existing queue-aware congestion avoidance schemes based on priority queue or FIFO scheduling methods fail to differentiate among several levels of severity in emergency packets, resulting in the delayed or dropped transmission of packets. To addresses the issue of handling the emergency packets in the high and low-priority queues, we propose a scheme called Dual Queue Aware Congestion Avoidance, which operates with a dual queue structure combined with an Emergency-aware Packet Placement Algorithm (EPPA). The scheme classifies packets into high-priority and low-priority queues according to sensor type and assigns an emergency flag to indicate the severity. Unlike conventional FIFO approaches, emergency packets are placed immediately after the currently processed packet to ensure timely treatment without disrupting the ongoing transmission. A large number of simulations are carried out using NS-2.35 on Ubuntu. The results show that D-QACA significantly reduces buffer loss probability and high-priority packet loss—achieving up to 80% lower buffer loss and 90% fewer emergency packet drops compared to state-of-the-art schemes such as QACA and DCCA. The scheme enhances performance through an emergency-aware repositioning mechanism that allows critical packets to be served after the packet currently in processing.

## Introduction

Internet of Healthcare Things (IoHT) comprises wearable and non-wearable devices, sensor/actuator circuits, wireless communication technologies, remote health monitoring, and IoT tools and applications [1,2]. IoHT builds an intelligent healthcare system to link numerous components to provide end users with a wide range of smart solutions [3]. IoHT supports human-machine interaction and real-time health monitoring systems, which increases patient involvement in decision-making. Significant benefits of IoHT applications include lower costs in healthcare systems, real-time emergency response, and remote monitoring in pandemic situations [4]. The advancement of IoHT is due to current hardware and software development for remote health, smart sensing, and medical surveillance. IoHT devices, may communicate with healthcare workers and medical professionals via online servers [5].

Three basic levels make up the IoHT system: i) The sensor layer, which includes various sensors like temperature, electroencephalogram (EEG), electrocardiogram (ECG), and photoplethysmogram (PPG). Individuals that are under observation have equipped with the variety of sensor circuits attached to the body of the patient. With the support of their connected or wireless communication utilities, the sensor nodes can detect vital signs. ii) The personal server layer, which has a body coordinator that can be turned on and off. As a central node, a coordinator node is the full control of a body’s sensors. It gathers sensed data and is a gateway to transmit it to other IoHT components. iii) The medial server layer, which includes medical practitioners and facilities. A fog or cloud computing node, database, monitoring tool, or secondary storage could all be found at the remote center. At this point, big data analysis starts if needed [6,7].

A huge quantity of health data is generated by IoHT which is stored at local or global cloud repositories that are accessible to hospital staff, doctors, and caretakers. It helps to analyze the symptoms, healthcare parameters monitored in the whole day, test reports, and medications to perform prediction, correlation, and categorization [8,9]. Smart devices communicate, transmit, and portray healthcare data via IoHT network. The network topology or heterogeneous computing framework are important element. Due to the massive number of sensing devices, it may generate network congestion, which results in packet loss, high energy usage, and low throughput. In an emergency scenario, a significant number of messages are transmitted, which might cause congestion. The problem gets worse if the sender is unaware of queue state of nearby nodes.

The most important issues regarding congestion management are still intense in IoHT. While existing congestion-aware schemes have managed to improve throughput and reliability, they still rely on FIFO scheduling and hence do not differentiate between packets according to their varying emergency severity. However, none of the existing studies address queue-level emergency processing, where urgent packets are dynamically sorted in the buffer to avoid loss or delay As a result, the packets that are critical and require priority transmission may be delayed behind routine monitoring data, compromising patient safety [10].

The motivation for this work stems from the crucial observation that the existing congestion-aware schemes in IoHT, such as QACA and DCCA, heavily rely on FIFO scheduling or static priority classifications, disregarding the varying severity levels of emergency packets. These limitations in emergency-driven healthcare can lead to intolerable delays or, worse, the loss of life-critical data, such as abnormal ECG or oxygen saturation signals. Timely and reliable delivery of packets containing emergency data directly impacts the survival of the patient; thus, there remains a need for a mechanism that can detect congestion and assign emergency packets a dynamic priority without interrupting ongoing transmissions. The proposed Dual Queue Aware Congestion Avoidance mechanism is motivated by this observation to minimize buffer loss, ensure minimal emergency delay, and maintain a fair mechanism for handling packets, while being lightweight on processing and flexible for modern IoT environments.

With the advent of IoHT, numerous smart devices are integrated with the patient’s body. It increases the number of requests to cause a bottleneck at intermediate devices to cause congestion. As a result, communication may be delayed and the most critical request may not be processed on time. The Q-based prioritizing mechanisms address the problem, however, the early detection of congestion is still challenging for the sender node as the state of the queue is not shared by the nearby nodes. Early identification of congestion awareness of surrounding nodes and dealing with the emergency packets on an urgent basis are challenging tasks in IoHT.

The major contribution of the article is as follows:

1. We explored the literature to identify a valid research issue for handling emergency packets in queue-aware congestion avoidance techniques in IoHT.
2. Next, a novel algorithm is presented to handle emergency data packets in IoT-based healthcare applications by categorizing them into high and low-priority queues based on their severity level. This contribution is significant in enhancing the efficiency and reliability of IoHT systems in emergency scenarios.
3. Finally, the proposed scheme is validated through extensive simulations to evaluate the performance of the proposed solution in contrast to dominating base schemes.

The remaining part of the manuscript is organized as follows; Section II explores the literature on packet drop and delay Issues in IoHT networks, preemptive, non-preemptive queues, and processing critical packets w.r.t health monitoring system. Section III presents the System Model and Problem Statement. Section IV explores simulation and results. Finally, Section V presents the conclusion and future extensions.

## Literature review

This section addresses current research activities on the topic of IoHT and its applications. The research community has recently shown a strong interest in IoT-based healthcare systems. In this way, congestion avoidance or management is a difficult study topic in IoHT. The following section looks at schemes that use a congestion-aware mechanism to communicate healthcare data.

In [11], the author discussed that the remote patient monitoring reduces hospital resource demands by enabling patients to stay at home and receive care. Data is transmitted via wearable sensors and smart devices into the cloud storage for insights and recommendations through machine learning algorithms. This approach faces challenges like security, congestion, and power management, making it less scalable. In [12], a Fog-based computing framework was designed to optimize IoHT by allowing local data processing, producing lower latency while improving responsiveness. The characteristics include distributed processing and scalability; these features generally make the computing framework fit real-time healthcare. However, the challenges of resource restriction, data security, and emergency data handling remain unresolved, which limits efficiency under heavy traffic conditions.

The authors of [13] review a variety of healthcare sensors with regard to their computing power, which is critical in IoHT. However, this study did not concern itself with emerging techniques such as machine learning, edge computing, and deep learning, which would offer better processing capabilities for healthcare data. The study in [14] focused more on the security and energy efficiency of wearable devices, particularly for short-range communication. Long-distance data transmission and the congestion of high data rates, however, remain unaddressed.In [15] discussed the Non-cooperative Gaming for Energy-Efficient Congestion Control (NGECC) technique that optimizes data transmission rates while avoiding parent node congestion. However, while this technique is efficient in the load balancing but the absence of packet-level prioritization makes it unsuitable for real-time emergency healthcare applications.

In [16], the author discussed the Congestion Control Algorithm (CCA) based on TCP, designed to adapt the congestion window to estimate bandwidth and round-trip time in IoT devices. In some points, it helps enhance throughput; however, it deteriorates under high RTT scenarios. The work in [17] addressed congestion by minimizing load on the constrained device by considering adaptation in rate-control and congestion detection under CoAP environments. The CoAR in [18] applied queue occupancy with adaptive buffer thresholds to minimize packet losses during heavy traffic. Reduction of congestion in CoAR remains dependent on generalized traffic prioritization while lacking mechanisms for dynamic differentiation for handling emergency packets. It identifies congestion based on queue occupancy. When a large number of packets must be transferred across the network, the proposed technique uses the adoptive buffer threshold technique to handle the situation and control the size of the queue.

The authors in [19] discussed the Priority Queue-based Token Bucket Algorithm (PQTBA) for reducing congestion in IoT networks. The proposed scheme used a discretionary rule, along with pre-emptive and non-preemptive techniques, for the prioritization of network traffic based on real-time requests. The PQTBA shows improved network throughput, packet loss ratio, and energy efficiency compared with baseline methods. However, the PQTBA relies primarily on bandwidth allocation and general traffic prioritization. Furthermore, it does not provide mechanisms to dynamically differentiate and reorder packets based on levels of urgency, which is very important for healthcare-directed IoHT systems. Hence, the PQTBA may still experience delayed treatment of life-critical information when congestion levels increase.

The Time Synchronized Channel Hopping algorithm (TSCH) discussed in [20] to detect and combat IoT congestion. By observing queue backlog conditions, nodes are capable of predicting congestion and switching to alternative parents and thereby getting rid of such potential bottlenecks. Although TSCH is a good approach for general IoT congestion management, it lacks packet-level priority, which is essential during emergencies and crucial for IoHT systems. These systems require that patient-critical data be sent with utmost urgency and minimal delay.

Mobile Edge Computing (MEC) frameworks, which process healthcare data near the source to reduce latency and alleviate congestion is discussed in [21]. Furthermore, particular attention has been gained by priority-based scheduling systems, especially those that prioritize tasks according to their levels of emergency, thereby ensuring that latency-sensitive healthcare data gets processed expeditiously. Most of these systems, however, focus on task-level priority assignment rather than on real-time queue-level packet management under congestion. The system of smart sensors and wearables, in combination with machine-learning methods such as Decision Trees and Naïve Bayes, was integrated into IoT systems emphasized in [22] to predict health risks and thus enable proactive care, especially beyond older patients. While this approach seems to accentuate the usefulness of AI-assisted IoHT, it concentrates mainly on health risk detection instead of congestion management and emergency packet prioritization.

The authors in [23] first examined the buffer overflow of 6LoWPAN-based IoT healthcare networks and developed an analytical model that calculates the probability of packet loss due to buffer overflow. The study depicts how congestion affects network performance and addresses the limitations of conventional congestion control methods for healthcare applications. The model presents a sound theoretical framework for assessing packet drop rates and buffer loss probabilities, but lacks an operational mechanism for the real-time, dynamic handling of emergency packets. As a result, although it provides insight into estimating congestion levels, it does not guarantee the timely processing of life-critical data.

The work by [24] incorporates the IoHT with resource-constrained medical devices, utilizing a 6LoWPAN protocol stack with a buffer-loss estimation model based on an M/M/1/K queue to manage congestion issues resulting from increased device activity. The theoretical model provides valuable information on a buffer-loss probability and delay of packets and denotes how the behavior seems improved when tested in constrained IoHT environments. However, while such work is beneficial for estimating congestion behavior, it falls short of providing a comprehensive solution for handling emergency packets in real-time queue operations. To enhance adaptability, [25] established an adaptive switching-based architecture of communication for the IoHT that activates dynamic switching between MQTT-SN and CoAP protocols in relation to network status. Simulation results verify enhanced throughput and reliability under high levels of traffic mediums. Although effective, this liberalizes protocol-based flexibility without addressing packet-level prioritization, which should have been involved, especially in life-critical scenarios where real-time processing of emergency data demands minimal delay.

In [26], the importance of proper scheduling for healthcare IoT systems is discussed, as such delays could lead to disastrous consequences, such as death. The authors proposed a Prioritized Scheduling (PS) scheme, derived from Earliest Deadline First (EDF), to mitigate such risk against congestion. While the PS scheme improves timeliness, it is, however, marred with complexity for implementation and overhead due to the process of packet classification. [27] investigated a congestion-aware data transfer mechanism utilizing hop-by-hop communication and the Analytic Hierarchy Process (AHP) for multi-parameter decision-making. Although it is efficient in choosing the best paths for packets, these schemes currently rely on routing-level congestion and lack fine-grained handling of emergency packets or speeds. In [28], a dynamic multi-level key management mechanism is proposed for homogeneous WSNs. This approach aims to enhance the security of resource-constrained networks by layering hierarchical keys in accordance with the network’s structure and communication. Although this framework does a great deal for authentication and resilience of keys in IoT environments, it primarily focuses on security and access control rather than congestion control or real-time prioritization of emergency packets.

The Distributed Congestion Control Algorithm (DCCA) was examined for IoT-enabled healthcare WSNs in [29], which combined priority-based routing with congestion control units towards an increase in reliability. While the performance of DCCA has been demonstrated for both loss reduction and network packet stability, DCCA is still not capable of prioritizing groups in a generalized way and does not differentiate between the severity of emergency cases within queues. The author in [30] discussed the queue-aware congestion avoidance (QACA) scheme, which uses controlled acknowledgment for congestion avoidance. The suggested framework shares the queue status with the sender nodes via a controlled acknowledging-based approach to reducing the congestion. When the queue reaches 50, 70, or 90 percent, the technique takes into account the three probable rates for transmitting the acknowledgment packet every 10, 5, or 1 packet. By adjusting the data rate, congestion can be promptly identified and potential congestion can be avoided. A main drawback of the QACA approach, is that it does not differentiate or prioritize emergency packets, leaving critical data susceptible to delay or loss under congested network conditions.

In [31], the author presented a lightweight hierarchical model using fuzzy logic and hop-by-hop encryption schemes. At the intra-cluster level, the cluster heads calculate a trust score for member nodes using fuzzy logic and accept data from only those trusted nodes. On the inter-cluster level, data is encrypted using a column transposition encryption algorithm, with the encryption keys updated dynamically at each hop to enhance resilience. The scheme demonstrates improvement in packet delivery ratio, throughput, and resistance to spoofing attacks compared to classical security mechanisms, as shown in the simulations. However, the method largely addresses the trust and security problems without directly addressing queue-level congestion and emergency packet prioritization, leaving a gap for real-time IoHT systems.

A dynamic trust-based clustering method for secure data gathering in IoT is discussed in [32]. The scheme evaluates nodes based on both direct, indirect, and historical trust, together with parameters such as energy, centrality, and neighbor count. Then, a genetic algorithm is used to optimize the selection of cluster heads, ensuring fault tolerance by having a backup cluster head. The model thus proved efficient in improving security and efficiency, but did not discuss any real-time congestion handling or scheduling for emergency packets, which are important in IoHT.

In [33], Mokhtari et al. have discussed a layered approach to control congestion for clustered IoT networks, utilizing both intra-cluster and inter-cluster approaches. The intra-cluster phase divides each node into nine states using the congestion score (CS) and buffer space (BES) for adaptive decision making. The inter-cluster phase utilizes waiting time to receive acknowledgment, a back-off timer, a sequence number, and a retransmission counter to control the communication of the cluster head. The scheme show improvements in the congestion score, packet loss, energy saving, and less end-to-end delay than those in conventional methods. However, it only emphasizes general congestion reduction without packet-level prioritization based on emergency severity, which is crucial in IoHT scenarios.

From the literature review, it is observed that most of the queue-based congestion schemes lack in handling the emergency packets as per the demand and processing the packets as per the sequence of arrival. Because of this, some scenarios lead to delays in the processing of the emergency packets [19,23,26,29,30]. In QACA, congestion avoidance is better than other schemes but it does not focus on processing the emergency packets. It is also mentioned that the delay in processing emergency packets in IoHT can have serious repercussions. In the literature, the base schemes categorize the packets into high and low priority but do not process them as per the emergency level of the packets.

## Materials and methods

### System model and problem statement

This section focuses on the system model, which incorporates a smart health monitoring system to ensure early detection of congestion in network queues, thereby preventing packet loss during emergency messaging. The study’s goal is to develop a priority-based queuing technique for IoT-based health monitoring systems that handles packets containing emergency data. During emergency scenarios, congestion occurs when a large number of messages are exchanged among nodes and the gateway. The model ensures the timely delivery of emergency packets even when congestion levels are increasing.

This system model consists of four types of nodes, including i) leaf nodes, which are sensing devices that include wearables and non-wearable devices. The aforementioned sensing devices are utilized to receive data from the body of the patient. ii) The collector node, whether a mobile or dedicated computational device, receives packets from the network and classifies them as high or low-priority packets as well as detects the severity of the packets. iii) intermediate nodes are devices that are medical objects and have substantial computational and network resources; the intermediate nodes process data that is obtained from the sensing devices based on the packet’s priority and forward to the local sink node for further processing iv) Local sink nodes are fog servers with vast computational power; the sink node’s primary function is to perform preprocessing on packets received from intermediate nodes, i.e., to determine the type of medical services required by the patient by analyzing packet data; and V) the root node, which is the cloud. The cloud is linked to the hospital administration system, which will be used by medical staff.

**Fig 1:**
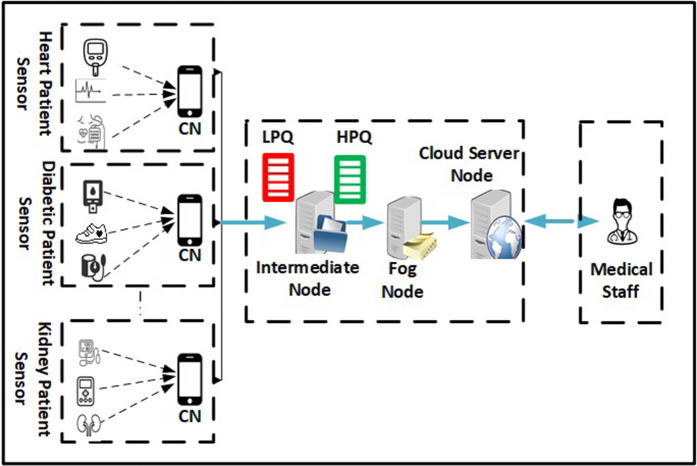
Proposed IoHT healthcare system architecture.

The IoHT sensing devices, including smartwatches, smartphones, and other gadgets, serve as a leaf node. Nowadays, electronic watches and smartphones are frequently used as leaf nodes to route the sensing data toward local sink nodes via collector and intermediary nodes. This processed information is transferred to the cloud servers (root nodes) via the network, as presented in the Fig 1.

The primary issue is that packets in both high-priority and low-priority queues are processed according to the first-in-first-out method. The existing approaches assume that packets in each queue are at a similar level and can be processed in the order of arrival, but the emergency factor is not considered. The Packets in both queues face different levels of emergency, which increases the chances of dropping off the emergency packets waiting in a queue due to the first-in-first-out approach. It also increases the waiting time for emergency packets, which in turn increases packet loss, delay, and congestion.

### Dual queue aware congestion avoidance scheme for emergent packet

This section describes the proposed “Dual Queue Aware Congestion Avoidance (D-QACA) scheme.” The suggested system employs a priority-based queue, with each node having two buffer queues. Each queue has a different priority level: low and high. When the node gets an input packet, it categorizes it and delivers it to its appropriate queues. The packets in the high- and low-priority queues are not at the same level and have different levels of emergency. For dealing with the emergency, the normal queue is replaced with the priority queue. In the priority queue, each packet is assigned priority according to the level of emergency. The packet containing the emergent data is assigned the highest priority and processed first if no packet is currently being processed. If any packet is currently processing, the packet is stored in the second place.

The suggested approach provides significant queue management benefits, particularly in scenarios where crucial data must be processed on time. Data packets are classified into high and low-priority queues based on their urgency, ensuring that the most vital information is handled first. The use of FIFO (First-In, First-Out) handling for packets with the same emergency level promotes fairness by processing packets sequentially. It eliminates bottlenecks and minimizes delays when processing crucial data. Furthermore, the technique dynamically modifies the queue, replacing less urgent packets with more vital ones as they arrive, thus improving the overall efficiency and responsiveness of the system.

The proposed approach is critical for healthcare applications, specifically real-time patient monitoring and emergency response systems. It allows for effective prioritizing of data from patient sensors, ensuring that vital alerts, such as a fast drop in oxygen levels or a spike in blood pressure, are handled first. The system ensures that the most essential cases are addressed quickly by arranging data into high and low-priority queues and then prioritizing based on the emergency level of the packets. This technique improves the responsiveness of remote patient monitoring, tele-medicine, and automated decision support systems, resulting in better patient outcomes and more efficient use of medical resources, particularly in high-stress scenarios such as emergency rooms or mass casualty incidents. The list of notations used in this work is shown in Table 1.

**Table 1:** List of Notations.

Sr.	Notation	Description
1	r-node	Root Node
2	inode	Intermediate Node
3	LPQueue	Low Priority Queue
4	HPQueue	High Priority Queue
5	lc_node	Lowest cost node
6	Packet	Packet Received from the root node

### Queuing at an intermediate node

The queues are maintained at the intermediate node, where packets are classified into high or low priority as per the nature of the sensor. These packets are processed as per the emergency level and priority assigned to the packets. Each packet is assigned an emergency flag (Eflag), where Eflag = 1 denotes emergency/life-critical data and Eflag = 0 represents normal data. The collector node sets this flag based on sensor type or abnormal readings. Packets containing sensitive data, such as data related to the heartbeat and brain, are placed in high-priority queues and processed according to the level of emergency. Packets containing less sensitive data are placed in a low-priority queue.

**Fig 2:**
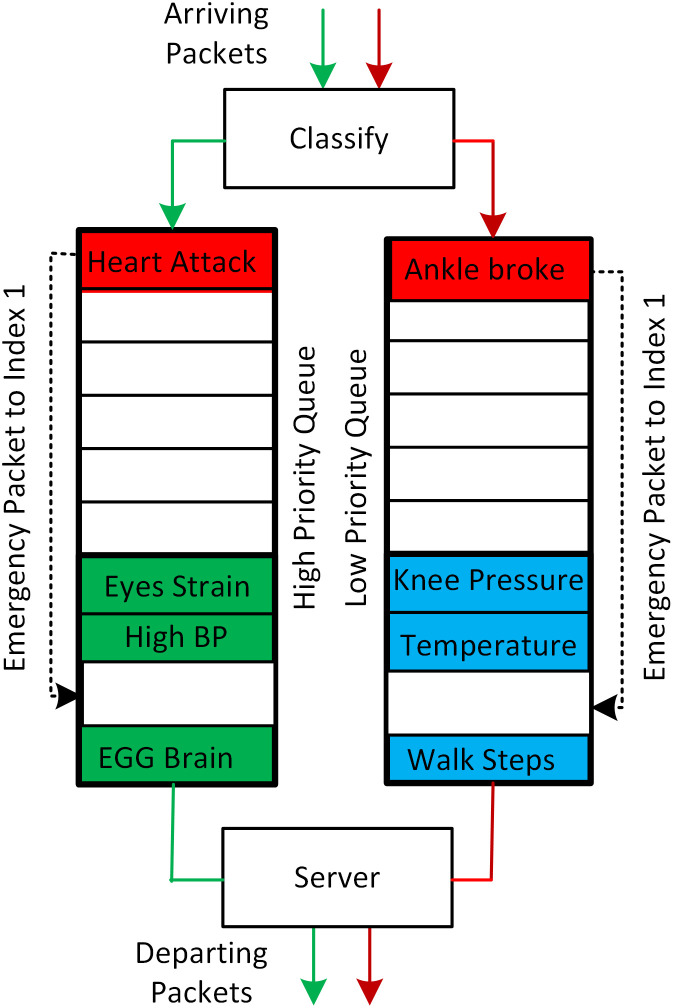
Queue management model for emergency packet placement.

The system implies that sensors generate patient data packets, which are then forwarded sequentially to a collector node; each packet is assigned priority and emergency level. The system has two queues, one for high-priority packets and one for low-priority packets, such that packets with higher urgency are handled first. If two packets have the same emergency level, the one that arrives first is prioritized. The system assumes that processing is non-preemptive, meaning that ongoing packet processing is completed before a new packet is processed. The proposed queue management model will dynamically adjust the high-priority packets by placing them at an index next to the packet currently in process, as illustrated in Fig 2. The model runs with a finite buffer capacity, highlighting efficient management to avoid overflow. These assumptions provide a realistic and solid foundation to assure system reliability in the context of healthcare.

### Emergency-aware packet placement algorithm

The D-QACA functionality is presented in Algorithm 1 titled Emergency-aware Packet Placement Algorithm (EPPA). EPPA employs an index+1 insertion policy, meaning that any arriving emergency packet is placed immediately after the currently processing packet in the queue, ensuring fast service without preemption. It focuses on managing the emergency packets by placing them at the front of the queue to manage the timely delivery of the packets without excessive delays. In steps (1–6), the Receive Packet function is responsible for managing incoming packets by determining their priority and placing them in the appropriate queue. When a packet is received, the function first checks its priority flag. If the flag indicates that the packet is of high priority, the packet is inserted into the High Priority Queue (HPQueue). If the packet does not have a high-priority flag, it is treated as a lower-priority packet and inserted into the Low Priority Queue (LPQueue). Once the packet is placed in the correct queue based on its priority, the function concludes its operation. This process ensures that high-priority packets are handled before lower-priority ones. Steps 7–17, the insertion queue function is designed to insert a packet into the appropriate position within a queue, which can be either a high-priority or low-priority queue. The function begins by initializing an index to zero. It then checks the size of the queue. If the queue is empty or has only one packet, the incoming packet is directly added to the queue, regardless of its emergency flag (Eflag). If the queue has more than one packet and the packet’s Eflag is 0, the packet is simply added to the end of the queue. However, suppose the queue contains more than one packet and the Eflag is set to 1, indicating a higher level of urgency. In that case, the packet is inserted at the second position in the queue. We represent the queue at any time as


$$\begin{array}{c} Q=[p_{1},\;p_{2},\;\ldots ,\;p_{L}]. \end{array}$$

When an emergency packet $p_{e}$ arrives, it is inserted at position 2:


$$\begin{array}{c} Q^{\prime }=[p_{1},\;p_{e},\;p_{2},\;\ldots ,\;p_{L}]. \end{array}$$

This operation corresponds to the non-preemptive “index+1” insertion rule. This approach ensures that packets with higher urgency are prioritized within respective queues.

**Algorithm 1: Emergency-aware Packet Placement Algorithm**

1  **Input:** Packet received from root node

2  **Output:** Acknowledge from each node

3  **Function** Receive_Packet(Packet):

4    **if** *Packet.flag == ‘High_Priority’* **then**

5      insertInQueue(HPQueue, Packet)

6    **end**

7    **else**

8      insertInQueue(LPQueue, Packet)

9    **end**

10 **End Function**

11 **Function** insertInQueue(Queue, Packet):

12   Set index = 0

13   **if** *Queue.size == 0* ***or*** *(Queue.size == 1* ***and*** *Packet.Eflag == 0* ***or*** *1)* **then**

14     Queue.insert.add(Packet)

15   **end**

16   **else if** *Queue.size > 1* ***and*** *Packet.Eflag == 0* **then**

17     Queue.insert.add(Packet)

18   **end**

19   **else if** *Queue.size > 1* ***and*** *Packet.Eflag == 1* **then**

20     Queue.insert.At(index+1, Packet)

21   **end**

22 **End Function**

## Results and discussion

This section provides the simulation results that are extracted for two different channel capacities of 120kbps and 250kbps. The residual queue for this analysis is set to 70, i.e., the acknowledgment is sent when the Queue is 70 percent full. A list of simulation parameters is shown in Table 2. To validate the performance of the proposed D-QACA, extensive simulation on NS2.35 is performed where the TCL file includes the nodes configuration, node deployment, message initiation, and trace annotation. NS-2.35 was selected because all baseline schemes, including QACA, DCCA, and the analytical model, were implemented and verified within it, ensuring method continuity. The study assumed that the intermediate node is responsible for transmitting the data to the sink node. The study also assumes that the maximum of three backouts represented by m and the re-transmissions n are allowed. The packet collision ratio is 0.10 and the number of leaf nodes is 2–10, the number of packets that can be sent is 10 while the packet transmission rate is 32 packets/ second. All the specifications are taken from the study presented in [23]. The study analyzed the packet drop probability due to the low buffer capacity, this is due to the low-priority packets having arrived at the buffer and there being no buffer space for the high-priority packets, the number of packets lost is also due to the overflow of the buffer and the total number of packet who reach the local sink node, the study considers the packets which have the highest priority. The base schemes for this work are the Analytical Model [23], DCCA [28] and QACA-70 [29]. The study used a channel capacity of 250 kbps and 120 kbps.The selected channel capacities correspond to the IEEE 802.15.4/6LoWPAN data rates widely used in IoHT deployments.

**Table 2:** Simulation parameters for proposed D-QACA.

Parameters	Values
Simulation Time	110 s.
Deployment Area	500 x 500 m.
Transmission Range	350 m.
Number of Nodes	50
Data Packet Size	500 bits
Number of CH	4
Number of Simulations	10
Time Consumed	0–20 seconds
Number of Packets	0–40 packets
Density	10–110 users
Control Message Size	100 bits
Duration of Data period	10 s
Chanal Capacity	150 kbps & 250 kbps
Sensing radius	20 m
Maximum Packet in Queue	50

### Buffer loss probability

**Fig 3:**
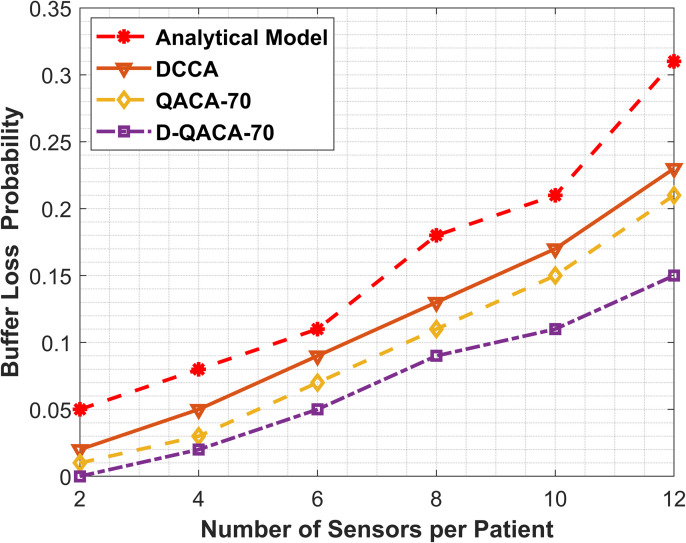
Buffer loss probability at leaf nodes with 250 kbps channel capacity.

In Fig 3, the Analytical Model and DCCA face notable buffer loss as the load increases, whereas QACA-70 exhibits a spike at load 6 whereas performs well otherwise. D-QACA-70 achieves the lowest buffer loss across all loads, demonstrating excellent performance for leaf nodes. When the number of sensors per patient is 6, the buffer loss probability is 0.11, 0.09, 0.07, and 0.05 for Analytical Model, DCCA, QACA-70, and D-QACA-70, respectively.

**Fig 4:**
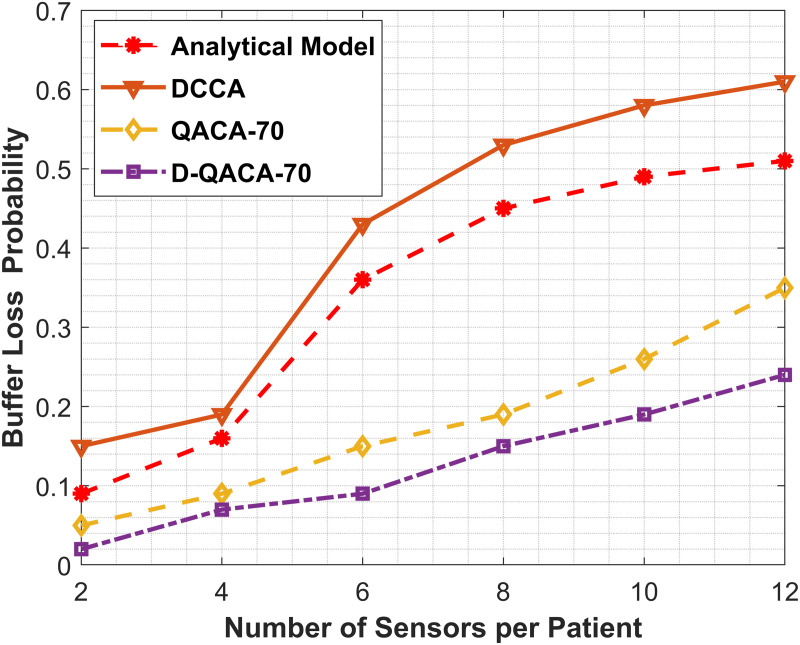
Buffer loss probability at intermediate nodes with 250 kbps channel capacity.

In Fig 4, higher buffer loss is observed in contrast to leaf nodes due to increased traffic at the intermediate node for 250 kbps. The buffer loss for the Analytical Model and DCCA is 0.36 and 0.46 when the number of sensors per patient is 6. In contrast, QACA-70 reduces to 0.15 while D-QACA-70 achieves the lowest probability of 0.09.

**Fig 5:**
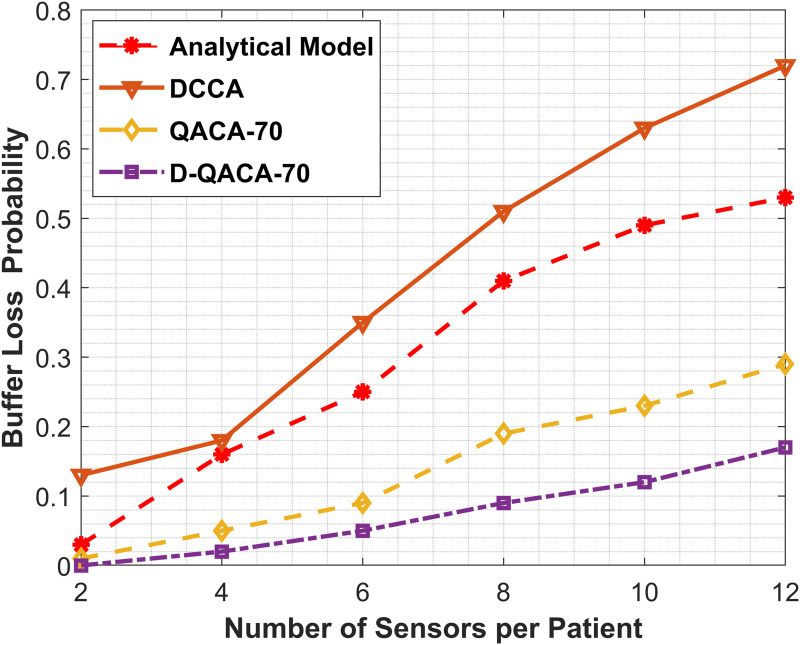
Buffer loss probability at leaf nodes with 120 kbps channel capacity.

At a reduced channel capacity of 120 kbps, Fig 5 illustrates buffer loss probability at the leaf node where the Analytical Model and DCCA show significant increases as 0.25 and 0.35 when the number of sensors per patient is 6. QACA-70 improves by 0.09 but the D-QACA-70 outperforms the by achieving 0.05.

**Fig 6:**
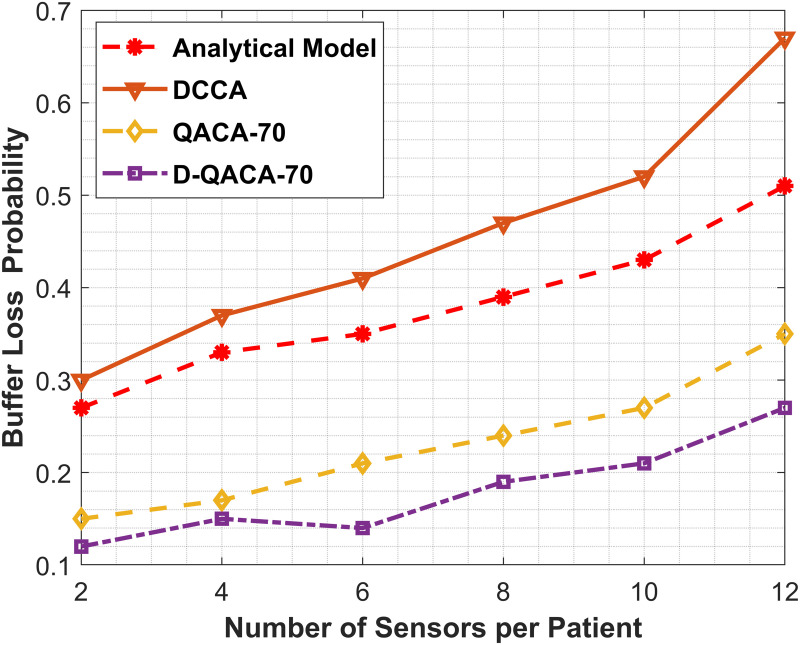
Buffer loss probability at intermediate nodes with 120 kbps channel capacity.

Fig 6 elucidates the buffer loss probability at intermediate nodes with 120 kbps channel capacity. The Analytical Model and DCCA exhibit considerable increases in buffer loss probability, although, QACA-70 and D-QACA-70 perform better. D-QACA-70 still has the lowest buffer loss at a higher rate.

It has been observed that the buffer loss probability for the proposed scheme for the emergency packets is almost zero even if the queue is 90% full at intermediate and leaf nodes. The packets are only dropped from the queue tail whereas the emergency packets are directly placed at the first index next to the current packet in the process.

### High-priority packet lost per second

**Fig 7:**
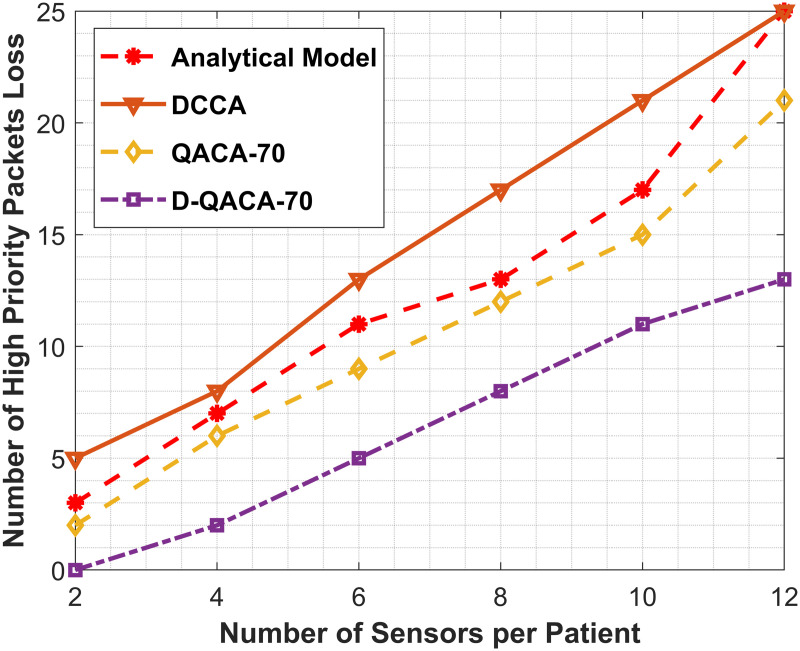
High-priority packet loss at leaf nodes with 250 kbps channel capacity.

For leaf nodes with a channel capacity of 250 kbps, the Analytical Model and DCCA suffer from the largest packet loss of 11 and 13 when there are 6 sensors per patient as shown in Fig 7. It increases rapidly as the load increases. These schemes handle congestion less efficiently, resulting in increased packet loss due to their inability to dynamically prioritize packets under high load. QACA-70 performs better with 8 packets lost whereas D-QACA-70 drops 5 packets only. This is due to D-QACA’s use of dynamic priority queuing, which effectively prioritizes high-priority packets and better manages queue congestion.

**Fig 8:**
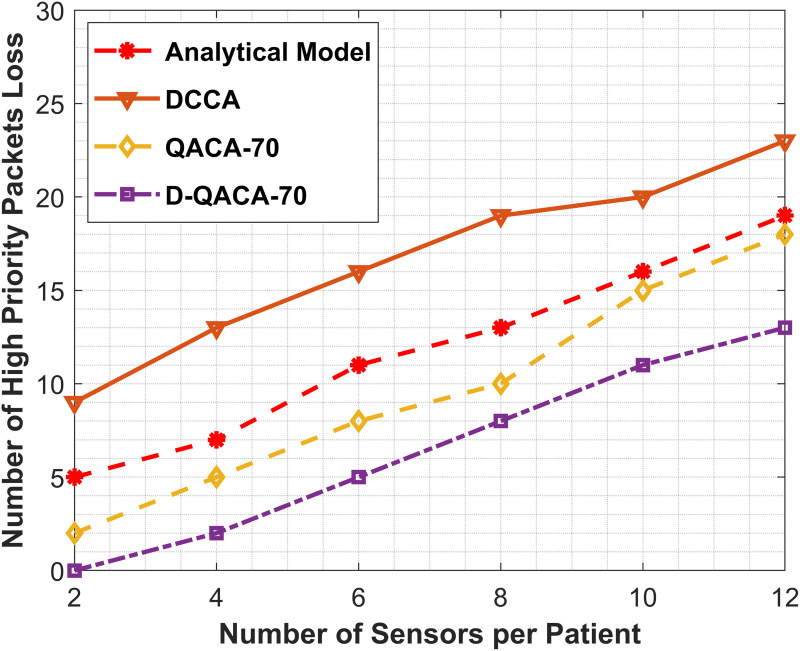
High-priority packet loss at intermediate nodes with 250 kbps channel capacity.

In Fig 8 for Intermediate Nodes with 250 kbps, the trend is similar. DCCA and the Analytical Model exhibit high packet loss whereas QACA-70 performs moderately and D-QACA-70 bears a low packet loss due to its adaptive nature which reacts to changing traffic situations.

**Fig 9:**
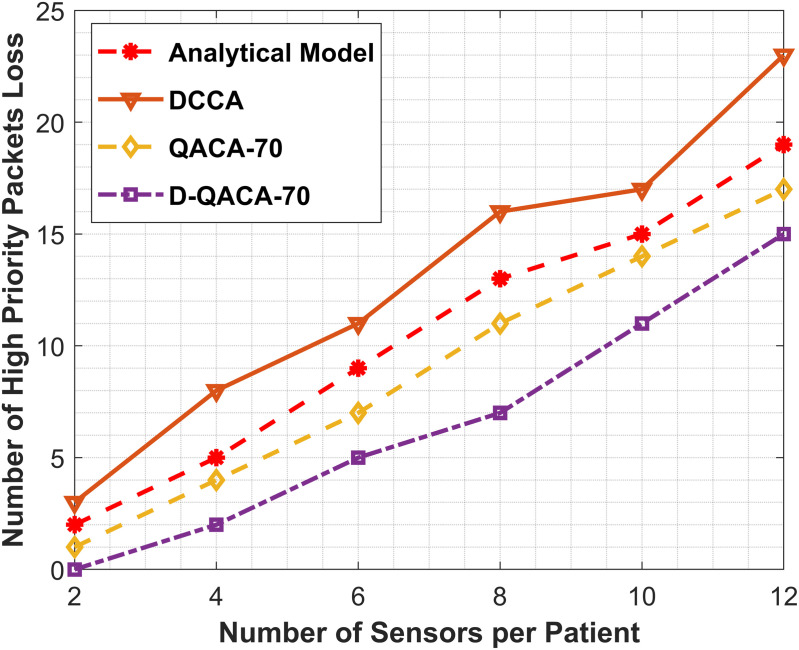
High-priority packet loss at leaf nodes with 120 kbps channel capacity.

Fig 9 shows that when the channel capacity drops to 120 kbps for leaf nodes, packet loss increases across all models due to bandwidth limitations. However, D-QACA-70 continues to outperform other schemes, showing better control of high-priority traffic by dynamically adjusting to buffer conditions and load. This is due to the selection procedure of the proposed scheme, D-QACA-70 classifies the packet and transfer to high or low priority queues for immediate processing based on the emergency. The incoming packet is placed in the next index of queue which reduces the chances of high priority packet loss due to “drop tail” is almost zero.

**Fig 10:**
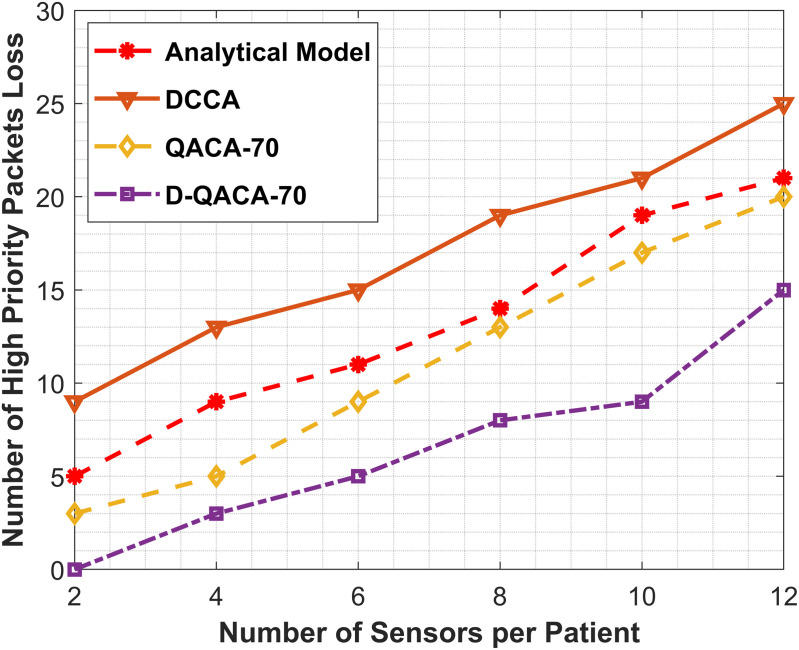
High-priority packet loss at intermediate nodes with 120 kbps channel capacity.

The number of high priority packets loss is 19, 21 and 17 when the number of sensors per patient are 10 while D-QACA-70 outperform other scheme by providing 9 high priority packets loss as shown in figure 10.

### Packets delay

**Fig 11:**
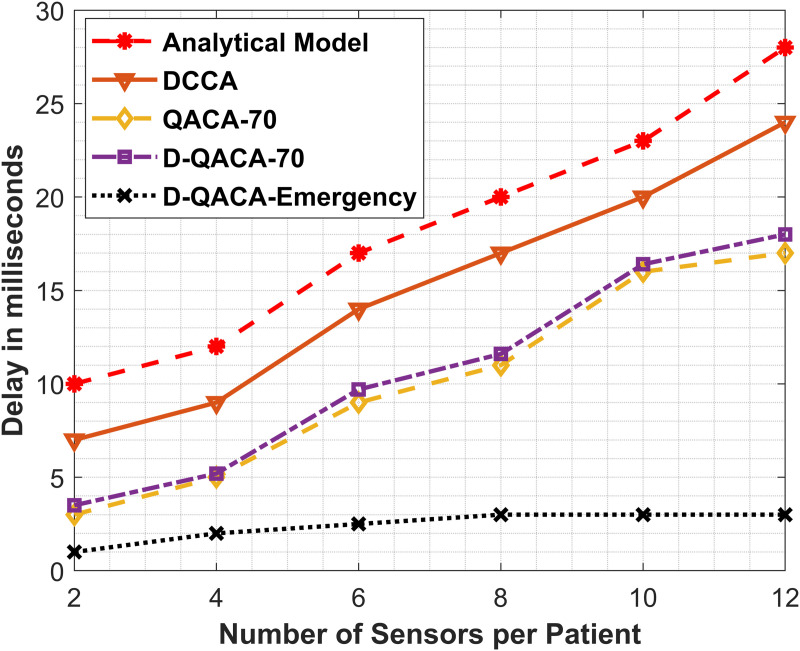
Packet delay comparison with 250 kbps channel capacity for 8 sensors per patient.

In Fig 11, the packet delay for the D-QACA-70 (Emergency) is 3 milliseconds, 20 milliseconds, 17 milliseconds, and 11 milliseconds for the Analytical model, DCCA, QACA-70, and D-QACA-70, respectively. The scenario is considered for 8 sensors per patient with a channel capacity of 250 kbps. The high value of D-QACA-70 can be due to an additional little computational cost in terms of performing emergency-related checks and swapping at index 1 or higher incrementally as per the existence of existing emergency packets but overall the delay of executing the emergency packets is much less as compared to other schemes.

**Fig 12:**
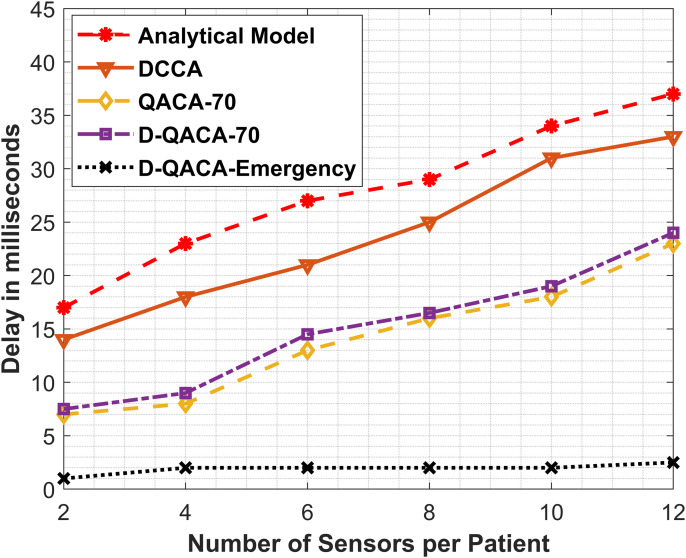
Packet delay comparison with varying sensors per patient at 250 kbps channel capacity.

Fig 12 elucidates the packet delay for the channel capacity of 250 kbps when the number of sensors per patient is varied from 2 to 12. For the 8 sensors per patient, the packet delay for the Analytical model, DCCA, D-QACA-70, and QACA-70 is 29 ms, 25 ms, 16.5 ms, and 16 ms, respectively whereas D-QACA-70 (Emergency) requires 2 milliseconds which is very less to manage the urgent scenarios.

## Conclusion

The study presents the D-QACA mechanism, developed to enhance congestion control in patient-centric IoHT systems, where emergency packets must be delivered with minimal latency and loss. The proposed scheme involves the use of a dual-queue architecture along with the Emergency-aware Packet Placement Algorithm (EPPA), an emergency flag (Eflag) that classifies and categorizes packets, and a non-preemptive index+1 insertion policy, which inserts critical packets at the position right after the packet that is currently being processed and at the same time maintains the current transmission. This placement scheme significantly increases reliability during congestion, lowering the likelihood of losing emergency buffer by 0.25 to 0.05 (an 80% relative reduction) and the likelihood of losing emergency packets by 10–1 (a 90 percent relative reduction). D-QACA can be highly beneficial for prioritizing essential medical traffic, even if it slightly increases the mean delay for regular packets. This trade-off is acceptable given the urgent nature of emergency data, which necessitates additional classification and placement processes. The current design, despite its advantages, fails to distinguish among the levels of emergency severity and focuses on buffer loss, delay, and the behaviour of emergency packets. Other performance indicators, such as energy consumption, jitter, fairness index, computational overhead, queue occupancy ratio, statistical variance, and large-scale scalability, were not explored. In addition, sensitivity and ablation studies were not included, which are essential for a more detailed characterization of performance. The future direction will focus on DQACA by adding multi-level categorization of emergency packet severity and by using lightweight machine learning techniques to recognize and rank emergency levels properly. We also plan to test the mechanism in more general IoHT systems, incorporate performance metrics, and test the scheme with modern simulation tools such as NS-3 or OMNeT++ to assess its applicability to real-world healthcare networks.
